# Innovative digital approaches to characterize core factors of patients with late-stage knee osteoarthritis: a cross-sectional study

**DOI:** 10.3389/fdgth.2025.1709182

**Published:** 2026-01-13

**Authors:** Marco Alessandro Minetto, Elisabetta Quilico, Federica Massazza, Gianmosè Oprandi, Chiara Busso, Giorgio Gasparini, Angelo Pietrobelli, John A. Shepherd, Steven B. Heymsfield

**Affiliations:** 1 Division of Physical Medicine and Rehabilitation, Department of Surgical Sciences, University of Turin, Torino, Italy; 2 Department of Orthopaedic and Trauma Surgery, Magna Græcia University, Mater Domini University Hospital, Catanzaro, Italy; 3 Postgraduate School of Orthopaedics and Traumatology, University of Turin, Turin, Italy; 4 Division of Orthopaedics, Koelliker Hospital, Turin, Italy; 5 Department of Surgical Sciences, Dentistry, Gynaecology and Paediatrics, Paediatric Unit, University of Verona, Verona, Italy; 6 Pennington Biomedical Research Centre, Baton Rouge, LA, United States; 7 Department of Epidemiology, University of Hawaii Cancer Center, Honolulu, HI, United States

**Keywords:** digital anthropometry, knee pain, knee osteoarthritis, pain drawing, physical performance

## Abstract

**Background:**

This study aimed to investigate in knee osteoarthritis patients the feasibility of a digital anthropometric approach for body size and composition assessment in combination with assessments of physical and pain characteristics.

**Methods:**

A convenience sample of 56 patients (34 females) was recruited. Clinical and radiographic evaluation, digital pain drawing and anthropometric assessments, and physical performance tests were performed.

**Results:**

Pain had an anterior distribution in all patients and several patients showed also a posterior and bilateral distribution. Median values of body fat percentage, fat mass index, and appendicular lean mass index were 28.3%, 7.8 kg/m^2^, and 8.4 kg/m^2^ in 19 males and 40.0%, 12.5 kg/m^2^, 6.8 kg/m^2^ in 28 females. Most of the patients had fat mass index higher than the cut-points for excess fat, while 2 male patients and none of the female patients had appendicular lean mass index lower than the cut-point for low mass. A relevant impairment of physical performance was observed in all patients.

**Conclusion:**

Innovative digital tools can be used to quantify the changes in body size and composition and the pain location and extension in patients with late-stage knee osteoarthritis.

## 1 Introduction

Knee osteoarthritis is not a single disease, but rather a heterogeneous condition presenting with different phenotypes characterized by variable underlying mechanisms, symptoms, and progression (1–6). Mechanistic, prescriptive, and prognostic phenotypes were previously described (on the basis of clinical, imaging, and biochemical marker measurements) to elucidate mechanisms of disease, identify patients more likely to respond to a specific intervention, and identify patients more likely to reach a specific outcome of interest (1–6). For example, the minimal joint disease phenotype can be identified through imaging markers assessing the radiographic severity, while the chronic pain phenotype and the metabolic syndrome phenotype can be identified through clinimetric (i.e., pain duration, intensity, and inference) and anthropometric assessments (7).

Advances in disease phenotyping can be obtained through both studies proposing new assessment methods and hypothesis-generating studies aimed to determine which characteristics identify patients in clinically important subgroups (8).

Recent technological advances made available for physicians new tools such as smartphone camera technologies and optical body scanners that capture a three-dimensional image of the body and provide useful biomarkers of body size, shape, and composition (9–11).

Recent experimental studies also showed that patient-reported pain location and extension through digital pain drawing may provide a clinically relevant characterization of pain pathophysiology (12, 13). In fact, an expanded distribution of pain was associated with signs of central sensitization in individuals with knee (14) and hip (15) osteoarthritis.

To our knowledge, no previous study was performed to phenotype knee osteoarthritis patients presenting with different disease severity with traditional physical performance tests in combination with innovative digital anthropometric and pain drawing assessments. Therefore, the primary aim of this study was to investigate the feasibility of a digital anthropometric approach for body size and composition assessment in knee osteoarthritis patients. The secondary study aim was to examine physical and pain characteristics in subgroups of knee osteoarthritis patients presenting with varying levels of perceived disability, in order to identify important attributes or problems that are common to different clinical phenotypes.

## 2 Methods

### 2.1 Participants and protocol

A convenience sample of 56 patients of both genders scheduled to undergo primary total knee arthroplasty participated in the study. Patients with primary knee osteoarthritis affecting the tibiofemoral and patellofemoral compartments were included. Exclusion criteria were: secondary osteoarthritis; previous knee joint replacement surgery or any other lower limb surgery (of the side affected by knee osteoarthritis) within the previous 6 months; presence of metabolic, neurological, or other severe medical conditions hindering the ability to participate in the study; presence of cognitive disturbances that could influence completion of self-administration questionnaires and pain drawings.

Clinical and radiographic evaluation, administration of outcome and pain questionnaires, anthropometric assessment, and physical performance assessments were performed in a single experimental session.

All patients gave their written consent after receiving a detailed explanation of the protocol. The study conformed to the guidelines of the Declaration of Helsinki and was approved by the local ethics committee (protocol n. 0065654).

### 2.2 Radiographic and clinical evaluation

All patients underwent weight-bearing, fixed flexion anteroposterior and lateral radiographs of the affected knee to evaluate the radiographic disease severity according to the Kellgren-Lawrence 0–4 grading scale (16). Moreover, weight-bearing anteroposterior radiographs of the entire lower extremities (“teleradiographs”) were taken in a standing position to assess in selected cases (see Results) the anatomical tibio-femoral angle (i.e., the angle between the anatomical axes of femur and tibia).

Osteoarthritis was diagnosed according to the American College of Rheumatology classification (17), defined by regular knee pain and either radiographic evidence of osteophytes or a combination of morning stiffness, crepitus, and age ≥50 years.

The Cumulative Illness Rating Scale (CIRS) was adopted to assess the comorbidities (18). Its principle is to classify comorbidities by organ system affected (14 disease categories are included) and rate them according to their severity from 0 to 4. Within each category, if two diseases are present, the disease with the highest severity is counted. The following two indices can be derived from the CIRS: (i) the severity index is the mean of the scores of the first 13 categories, excluding psychiatric; (ii) the comorbidity index is calculated as the number of categories with a score of 3 or greater, including psychiatric.

### 2.3 Clinimetric assessments of patient characteristics

The following patient characteristics were investigated: pain phenotype measures (duration, intensity, interference, extension and location, number of pain areas), analgesic medication use, level of perceived disability, physical function, and quality of life.

Pain duration was queried using a 6-interval scale (less than 1 month, 1–3 months, 3–6 months, 6–12 months, 1–2 years, more than 2 years).

Pain intensity and interference assessment was performed with the Italian version (19) of the Brief Pain Inventory (20) that quantifies pain intensity with 4 items (worst, least, average, and right now), activity pain interference with 3 items (general activity, walking, work), and affective pain interference with 4 items (mood, relation with other people, sleep, enjoyment of life). All items are scored using an 11-point numerical rating scale (with 0 corresponding to “no pain”/“no interference” and 10 corresponding to “the worst imaginable pain”/“maximal interference”).

Pain extension and location were assessed through a user-friendly digital device featuring body charts templates, as previously described (12, 14). Frontal and dorsal body chart views were adopted: patients were instructed to colour every part of the affected lower limb where they perceived pain, regardless of pain type and severity. Pain extension was quantified (using custom software written in Matlab: image dimensions in height × width were 3,506 × 2,480 pixels) as the total number of pixels coloured within the anterior and posterior body chart perimeter, while pain location was assessed through a pain frequency map. Briefly, the pain drawings of all patients were superimposed to obtain a map with different colors indicating different percentages of patients reporting pain in a specific area (the lower limb of the right side was arbitrarily selected for all patients to represent the pain frequency map—see Results).

The number of pain areas was obtained from a pre-appointment health questionnaire, using a figure of the human body where patients were instructed to mark all the painful areas. For calculating the sum of the pain areas, the figure was divided into the following 19 subareas: back of the neck, upper back, lower back, chest, abdomen, left and/or right side of the jaw, shoulder girdle, upper arm, lower arm, hip (trochanter included), upper leg (knee included), lower leg (ankle and foot included).

Musculoskeletal pain can be defined widespread if it is present in at least 4 of the following 5 regions: left upper limb, right upper limb, axial region with the exclusion of chest and abdomen, left lower limb, right lower limb (21).

Patients were asked to indicate their preferred analgesic treatment (used as needed) and to rate its pain-relieving effect on a 4-point Likert scale with the following response options: 0, no effect; 1, mild effect; 2, moderate effect; 3, strong effect.

Patients were asked to complete the following self-administration questionnaires in a standardized order: 12-item Short Form survey (22); 8-item short form of the Neuro-QoL Lower Extremity Function—Mobility questionnaire (23); Lequesne algo-functional scale (24); functional score of the 2011 Knee Society scoring system (25, 26).

The 12-item Short Form Survey (SF-12) is a self-reported outcome questionnaire assessing the following eight health domains: physical functioning, role-physical, bodily pain, general health, vitality, social functioning, role-emotional, and mental health. Each domain score contributes to the physical and mental component scores.

The 8-item Neuro-QoL questionnaire investigates the lower extremity function for eight different activities of daily living (e.g., getting on and off the toilet, getting out of bed into a chair, walking for at least 15 min). All activities are scored using a 5-point numerical rating scale (with 1 corresponding to “unable to do” and 5 corresponding to “able to do without any difficulty”): the global score ranges from 8 to 40 and can be rescaled in a standardized score (T-score) to compare the value of a single patient to a general population reference sample (with a mean of 50 and a standard deviation of 10).

The Lequesne algo-functional index (ranging from 0 to 24) is related to pain or discomfort, maximum distance walked, and activities of daily living. Index score allows to distinguish among mild disability (between 1 and 4), moderate disability (between 5 and 7), severe disability (between 8 and 10), very severe disability (between 11 and 13), and extremely severe disability (≥14).

The 2011 Knee Society functional abilities score (ranging from 0 to 100) is related to: (i) walking and standing, (ii) standard activities of daily living, (iii) advanced activities, (iv) discretionary activities. Cut-off values of 85, 73, and 56 enable to distinguish among excellent, good, fair, and poor function (27).

### 2.4 Anthropometric and body composition assessments

Body weight and height were assessed with each patient in undergarments and barefoot. Body weight and height were measured (to the nearest 0.1 kg and 0.5 cm, respectively) using a standard scale with stadiometer (model Seca 799, Seca GmbH & Co. KG, Hamburg, Germany).

Optical images were taken with Mobile Fit app (version 3.0, Size Stream LLC, Cary, NC, USA) installed on an iPad (Apple Inc, Cupertino, CA, USA), using a standardized positioning protocol, as previously described (28–31). Voice commands from the app guided each patient into position for the self-scan as they assumed a “front A-pose” to capture the frontal image. Next, the patient was asked to assume a “side pose” to capture the lateral image. After the image capture, the app software generated a de-identified tri-dimensional humanoid avatar with associated anthropometric measurements and body composition estimates. The acquisition of the frontal and lateral images was performed in duplicate to obtain two avatars for each patient. The Mobile Fit app report includes whole-body and segmental circumferences, lengths, surface areas, volumes, and body composition estimates. The following body size, shape, and composition variables were considered (data obtained for the two avatars were averaged): body mass index, waist circumference, body fat percent, fat mass index and fat free mass index (i.e., fat mass and fat free mass scaled to height squared, respectively), appendicular lean mass index (i.e., sum of the soft lean tissue in the arms and legs scaled to height squared). The equations adopted to estimate body fat percent and appendicular lean mass are reported in Supplementary Table S1 (32, 33).

### 2.5 Physical performance assessments

The following performance-based tests of physical function (included in the minimum core set recommended by OARSI) (34) were performed: (i) 30-s chair stand test: patients were asked to stand and sit from a chair as many times as possible for 30 s (with their arms crossed over their chest) and the number of repetitions was recorded; (ii) stair climb test: patients were asked to ascend and descend a flight of 12 steps and the total time required to complete the trial was recorded; (iii) 40-m fast paced walk test: patients were asked to walk 4 times over a 14-m walkway at fast speed. A photoelectric system (Witty Gate, Microgate, Bolzano, Italy) was used to time the patients as they walked over the central 10 m of the walkway, excluding the initial 2 m and final 2 m, to allow for acceleration and anticipatory deceleration. The total distance covered (40 m) was divided by the total time taken to complete the 4 repetitions to obtain the average fast walking speed (m/s).

### 2.6 Statistical analysis

The Shapiro–Wilk test for normal distribution of the data failed, therefore non-parametric statistical tests were used. K-means algorithm cluster analysis was adopted to classify patients into the low and high severity subgroups: 20 cases in each subgroup are recommended for a reliable clustering (35), thus only two clusters could be identified given the sample size of our study. Cluster analysis was performed for each of the two gender groups on the following variables: Lequesne index, lower extremity function T-score, Knee Society functional abilities score, activity pain interference score, affective pain interference score. These variables were selected because they are commonly adopted in the clinical practice for patient phenotyping.

The Mann–Whitney *U* test was adopted for comparisons between different subgroups. Data were expressed as median and 1st—3rd quartiles.

The threshold for statistical significance was set at *P* = 0.05. Statistical tests were performed using the SPSS v. 20.0 (SPSS Inc., Chicago, IL, USA) software package.

## 3 Results

Fifty-six patients [22 males and 34 females, median (1st–3rd quartile) age: 77.3 (73.4–82.0) years and 72.2 (69.0–77.6) years, respectively] were evaluated. Their radiological and clinical characteristics are detailed in Table 1: all patients showed a moderate or severe osteoarthritis (13 male and 22 female patients had a Kellgren-Lawrence grade 3 and 9 male and 12 female patients had a grade 4) and most of them had both a Knee Society functional abilities score indicating poor function (18 out of 22 male patients and 34 out of 34 female patients) and a Lequesne score indicating a disability from very severe to extremely severe (11 out of 22 male patients and 24 out of 34 female patients). Moreover, all patients showed a below-average lower extremity function (median T-score: 36.8 in male patients and 34.9 in female patients) and a below-average physical health-related quality of life (median value of the physical component score: 36.8 in male patients and 30.9 in female patients).

**Table 1:** Median (1st–3rd quartile) values of the results of the radiological and clinimetric assessments obtained in the whole group of 56 patients (22 males and 34 females) and of physical performance assessments obtained in 18-22 males (22 males completed the 40-m fast paced walk test; 20 males completed the stair climb test; 18 males completed the 30-s chair stand test) and 32 females (two female patients were unable to complete the three physical performance tests).

Variable	Males	Females
Kellgren-Lawrence grade		
3: *n* (%)	13 (59.0)	22 (64.7)
4: *n* (%)	9 (41.0)	12 (35.3)
Knee Society functional abilities score	35 (18–50)	27 (16–41)
Lequesne index	11.5 (9.0–14.5)	13.0 (10.5–16.0)
Lower Extremity Function		
Raw score	27.0 (20.0–34.0)	24.5 (21.3–28.8)
T-score	36.8 (31.5–42.8)	34.9 (31.9–38.1)
12-item short form survey		
Physical component score	36.8 (24.6–40.5)	30.9 (27.9–36.4)
Mental component score	55.2 (46.8–58.7)	47.0 (36.9–53.8)
Fast walking speed (m/s)	1.27 (0.99–1.40)	1.16 (0.96–1.35)
Stair climb test (time—s)	16.1 (15.2–29.8)	25.6 (18.4–39.1)
30-s chair stand test (repetitions)	10 (7–11)	9 (7–11)

Two female patients were unable to complete the three physical performance tests, two male patients were unable to complete two of the three tests (i.e., the 30-s chair stand test and stair climb test), and other two male patients were unable to complete the 30-s chair stand test. Both groups of patients showed low walking speed (that resulted more than two standard deviations below the average values of age- and gender-matched healthy subjects for all patients) (34, 36), low stair climb test performance (i.e., the stair climb test time was more than two standard deviations above the average values of gender-matched healthy subjects for 19 out of 22 males and 31 out of 34 females) (34), and low 30-s chair stand test performance (i.e., the number of chair stand repetitions was lower than the 25th percentile of the normative distributions of age- and gender-matched healthy subjects for 18 out of 22 males and 22 out of 34 females) (34, 37).

**Figure 1:**
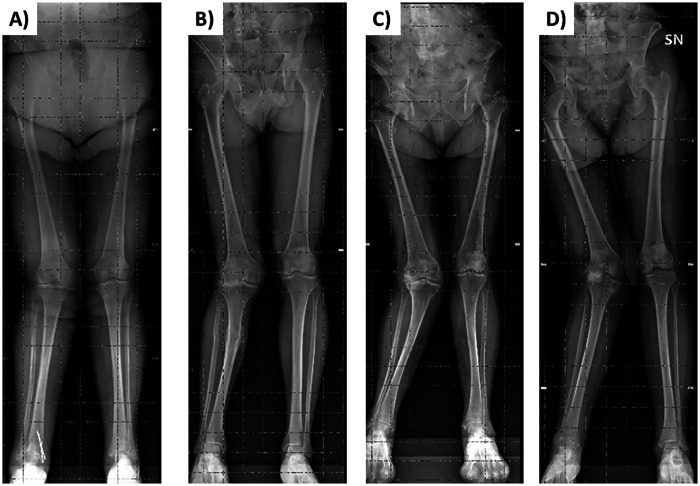
Weight-bearing anteroposterior radiographs of the entire lower extremities of 2 patients presenting grade II valgus deformity [tibiofemoral alignment for the right side of 11 ° in panel **(A)** and 13 ° in panel **(B)**] and of 2 patients presenting grade III valgus deformity [alignment for the right side of 22 ° in panel **(C)** and 25 ° in panel **(D)**].

**Figure 2:**
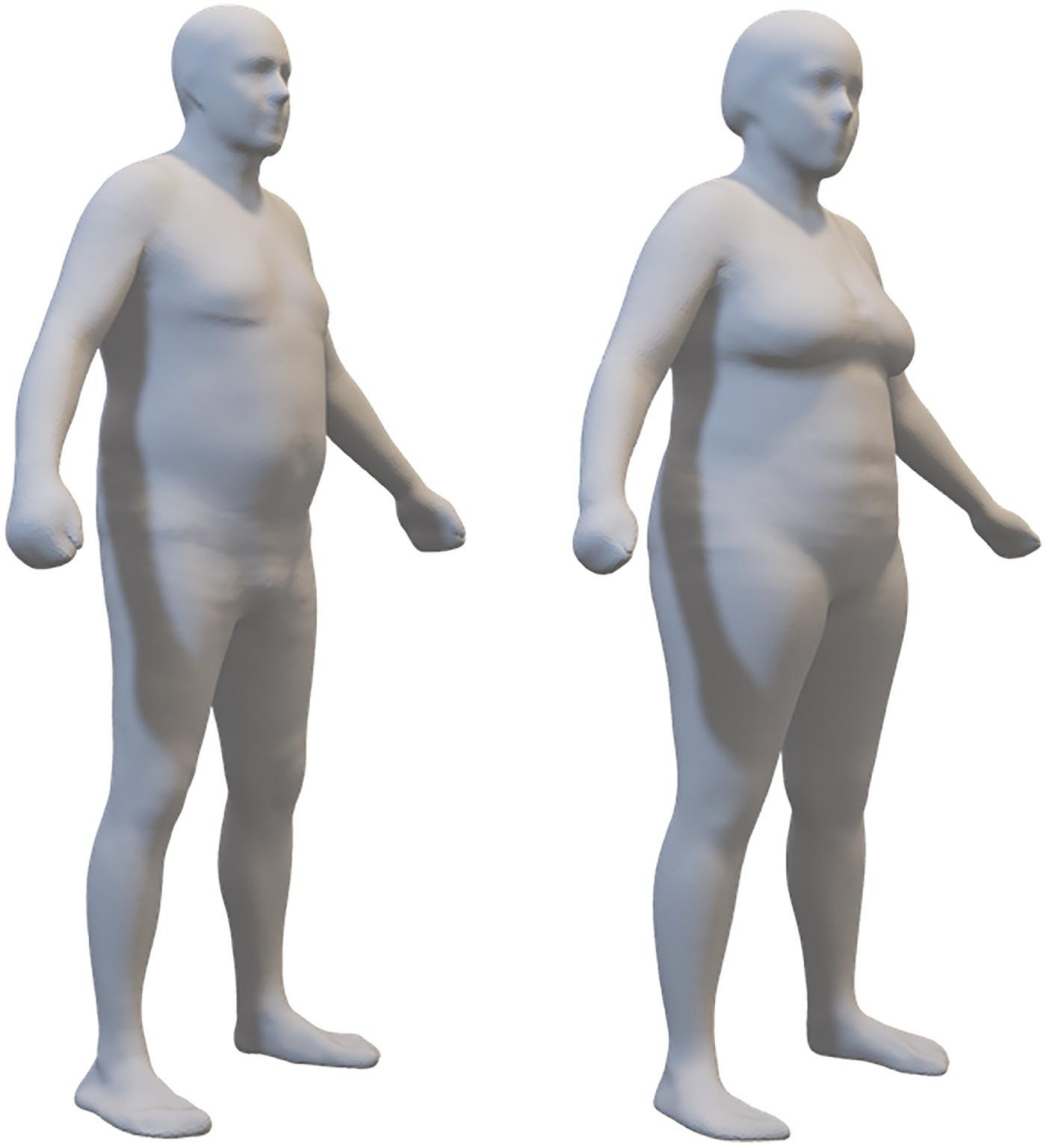
The average body shape of 19 males and 28 females in the sample. These 3-dimensional meshes are the sex-specific means of the registered meshes fitted to participant scans.

**Figure 3:**
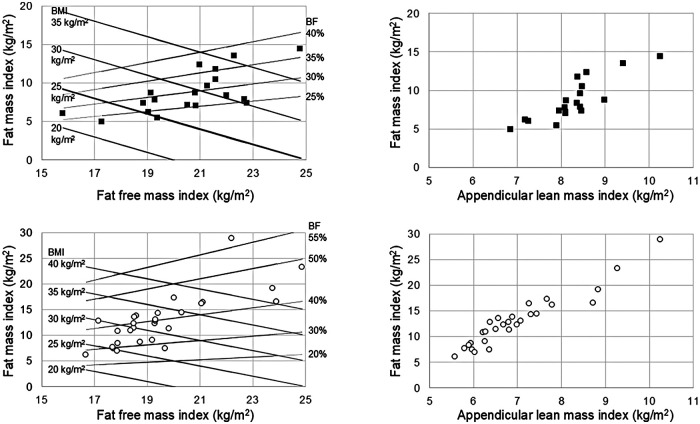
Body composition charts obtained in 19 male patients (upper panels) and 28 female patients (lower panels). Lines representing body mass index (BMI: continuous black lines) and body fat percentage (BF%: dotted black lines) are included in the left panels.

Frontal and lateral images were acquired in duplicate in all patients (scan time was around 30 s for each of the two acquisitions that were well tolerated by the patients), with the exception of 3 patients who were unable to maintain the standing position during image acquisition. Moreover, the frontal image could not be acquired in 2 patients presenting grade II valgus deformity (tibio-femoral angle between 10 ° and 20 °) and in 2 patients presenting grade III valgus deformity (angle > 20 °) (Figure 1). In addition, processing errors implied the image rejection for 2 patients: overall, body scans were not available for 9 patients (3 males and 6 females) and a total of 47 patients (19 males and 28 females) were evaluated (no statistically significant differences were observed between the two subgroups of 3 and 19 males and between the two subgroups of 6 and 28 females in age, body mass index, physical performance variables, and clinimetric variables of quality of life, comorbidities, pain intensity, pain interference: *P* > 0.05 for all the comparisons). Their average body shapes are shown in Figure 2 and their anthropometric and body composition characteristics are reported in Table 2. Body composition charts (38) obtained for male and female patients showed that the higher the fat mass index, the higher both the fat free mass index and the appendicular lean mass index (Figure 3): 10 out of 22 males and 21 out of 34 females had a body mass index ≥30 kg/m^2^, 14 out of 19 males and 24 out of 28 females had body fat >25% and >30%, and 16 out of 19 males and 21 out of 28 females had fat mass index higher than the cut-point for excess fat (>6 kg/m^2^ for males and >9 kg/m^2^ for females, respectively) (39). Moreover, 6 out of 19 males and 26 out of 28 females had waist circumference ≥102 cm and ≥88 cm (40), respectively, while 2 male patients and none of the female patients had appendicular lean mass index lower than the cut-point for low mass (<7.26 Kg/m^2^ for males and <5.45 Kg/m^2^ for females) (41).

**Table 2:** Median (1st–3rd quartile) values of the results of the body mass index and pain phenotype assessments obtained in the whole group of 56 patients (22 males and 34 females) and of the waist circumference and body composition assessments obtained in 19 males and 28 females.

Variable	Males	Females
Body mass index (kg/m^2^)	29.8 (27.2–30.9)	31.2 (28.3–36.5)
Body fat (%)	28.3 (25.2–32.3)	40.0 (32.5–42.9)
Fat mass index (kg/m^2^)	7.8 (7.1–10.0)	12.5 (8.9–14.8)
Waist circumference (cm)	96.4 (93.4–103.6)	110.4 (98.7–117.1)
Appendicular lean mass index (kg/m^2^)	8.4 (8.0–8.5)	6.8 (6.3–7.3)
Pain duration		
>2 years—*n* (%)	14 (63.6)	32 (94.1)
1–2 years—*n* (%)	3 (13.6)	2 (5.9)
6–12 months—*n* (%)	4 (18.2)	
3–6 months—*n* (%)	1 (4.6)	
Pain intensity (a.u.)	4.0 (2.4–5.5)	3.6 (2.8–5.0)
Affective pain interference (a.u.)	3.8 (1.6–6.3)	4.5 (3.4–5.7)
Activity pain interference (a.u.)	7.0 (5.1–8.2)	7.3 (6.1–8.0)
Bilateral knee pain—*n* (%)	11 (50%)	24 (71%)
Number of pain areas	3.0 (2.0–3.0)	3.0 (2.3–4.0)

Table 2 also shows the pain characteristics of the whole group of 22 male and 34 female patients: pain duration exceeded two years in the majority of the male patients and in nearly all female patients. Pain intensity and affective interference were between mild and moderate in most cases, whereas pain activity interference was predominantly rated as moderate to severe. Half of the male patients and more than two-thirds of the female patients showed a bilateral knee pain, while the median number of pain areas was 3 in both groups (no patients reported ≥4 painful areas in the 5 body regions). Half of the male patients (11 out of 22) and three-fourths of the female patients (26 out of 34) used analgesic drugs as needed with a referred pain relief from mild (3 out of 11 males and 7 out of 26 females) to strong (6 out of 11 males and 5 out of 26 females).

**Figure 4:**
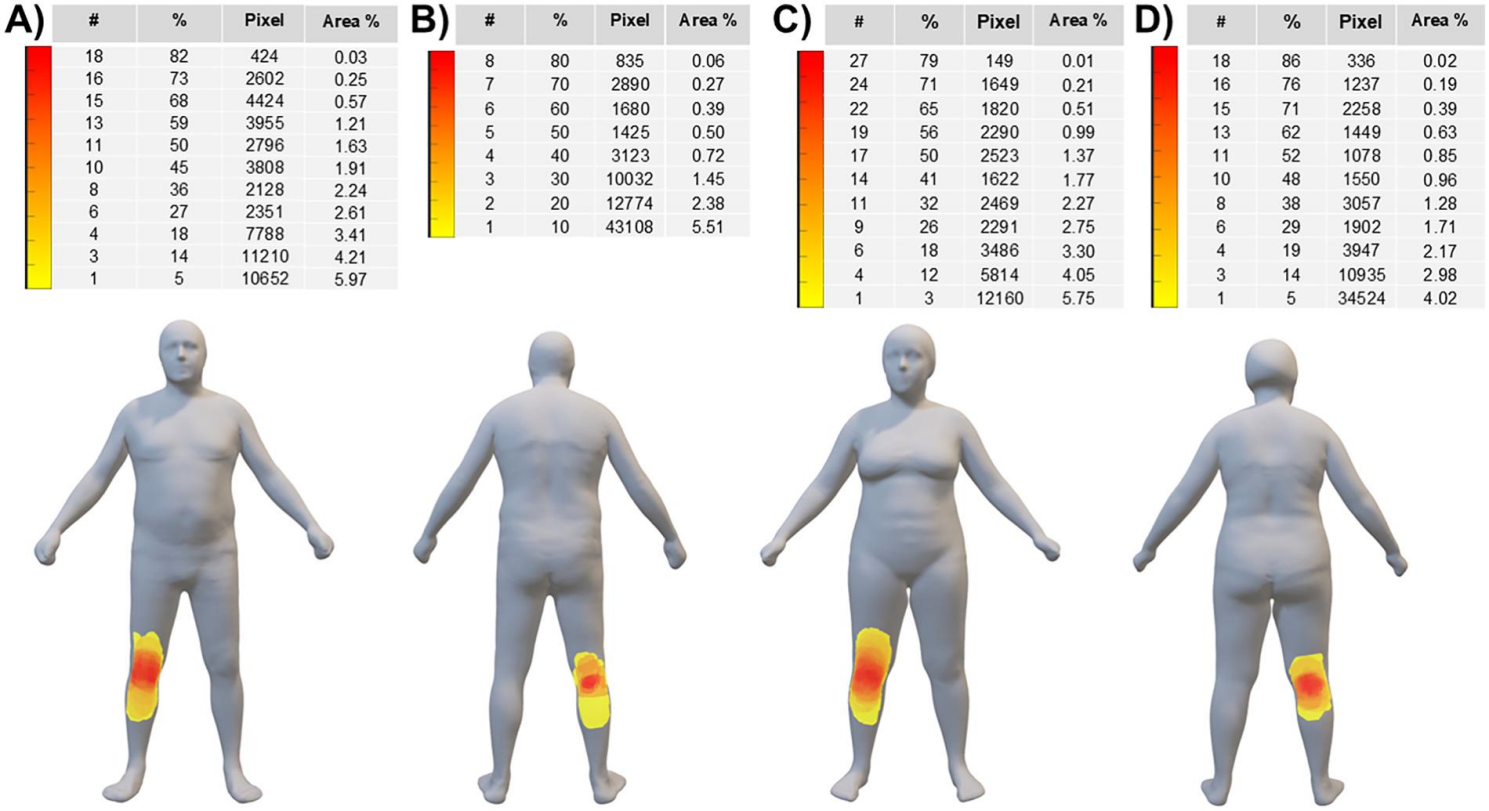
Pain frequency maps (reported on the mean avatars) for the whole group of 22 male patients (panels **A-B**) and 34 female patients (panels **C-D**). The color bar represents the frequency of colored areas (i.e., different colors indicate different percentages of patients reporting pain in a specific area): red and yellow areas represent, respectively, the most and less frequently reported areas of pain. #: number of patients; %: percentage of patients; Pixel: cumulative sum of pixels; Area %: extension of the painful area normalized to the anterior or posterior body surface area.

Figure 4 shows the pain frequency maps reported on the mean avatars for the whole group of 22 male patients (panels A-B) and 34 female patients (panels C-D): pain had an anterior distribution in all patients, while 10 males and 21 females showed also a posterior distribution. The median pain extension was 26,374 (11,620–43,167) pixels across the entire sample, whereas it was 19,727 (13,032–39,674) pixels and 27,853 (10,028–43,295) pixels for male and female patients, respectively.

K-means cluster analysis of the clinimetric scores identified two subgroups of patients herein referred to as low (10 males and 17 females) and high (12 males and 17 females) knee osteoarthritis severity. The Supplementary Figure S1 shows the bar charts of the final cluster centres that were adopted to visually inspect the separation of the clusters. According to the ANOVA table of the K-means cluster analysis (Table 3), all variables were significant in creating the clusters in male patients and three of the five variables were significant in female patients.

**Table 3:** ANOVA table of the K-means cluster analysis.

Male patients (*n* = 22)						
Variable	Cluster		Error		F	Sig
	Mean square	df	Mean square	df		
Lequesne index	278.850	1	9.783	20	28.505	0.000
Lower extremity function T-score	719.483	1	12.227	20	58.842	0.000
Knee Society functional abilities score	5,856.297	1	96.603	20	60.622	0.000
Activity pain interference score	55.274	1	2.759	20	20.035	0.000
Affective pain interference score	91.989	1	4.111	20	22.379	0.000
Female patients (*n* = 34)						
Lequesne index	365.654	1	8.815	32	41.480	0.000
Lower extremity function T-score	257.675	1	21.770	32	11.836	0.002
Knee Society functional abilities score	6,196.500	1	71.092	32	87.162	0.000
Activity pain interference score	8.905	1	2.992	32	3.047	0.090
Affective pain interference score	8.701	1	4.779	32	1.821	0.187

The *F* tests should be used only for descriptive purposes because the clusters have been chosen to maximize the differences among cases in different clusters. The observed significance levels are not corrected for this and thus cannot be interpreted as tests of the hypothesis that the cluster means are equal.

As shown in Table 4, the two subgroups were comparable for age and comorbidities in males and for comorbidities in females, while females in the high severity subgroup were older than those in the low severity subgroup.

**Table 4:** Median (1st–3rd quartile) values, mean differences and 95% confidence intervals of the differences for the results of the clinimetric, pain phenotype, anthropometric, body composition, and physical performance assessments obtained in the two subgroups of patients with low and high severity of knee osteoarthritis. Clinimetric, pain phenotype, and body mass index values were obtained from 27 patients (10 males and 17 females) with low severity and 29 patients (12 males and 17 females) with high severity of knee osteoarthritis. Waist circumference and body composition values were obtained from 25 patients (8 males and 17 females) with low severity and 22 patients (11 males and 11 females) with high severity of knee osteoarthritis. Physical performance values were obtained from 27 patients (10 males and 17 females) with low severity and 27 patients (12 males and 15 females) with high severity of knee osteoarthritis.

Variable	Subgroups	Low Severity	High Severity	*P* Value	Mean Difference	95% CI of the Difference
Gender distribution	Males	10/22	12/22	-	-	-
	Females	17/34	17/34	-	-	-
Age (years)	Males	76.9 (74.9–80.9)	77.3 (71.8–86.2)	0.77	−1.2	−8.0, 5.5
	Females	69.1 (68.0–72.9)	76.7 (72.1–79.9)	**0.001**	−6.6	−10.5, −2.8
CIRS—Severity index	Males	1.6 (1.3–1.6)	1.6 (1.5–1.7)	0.65	−0.08	−0.2, 0.1
	Females	1.5 (1.4–1.7)	1.5 (1.4–1.6)	0.46	−0.03	−0.2, 0.1
CIRS—Comorbidity index	Males	3.0 (1.0–3.0)	2.5 (2.0–4.0)	0.51	−0.4	−1.6, 0.7
	Females	2.0 (2.0–3.0)	3.0 (2.0–4.0)	0.17	−0.6	−1.3, 0.2
12-item SF survey—PCS	Males	41.1 (37.3–44.9)	26.4 (20.8–36.4)	**0.002**	13.2	6.4, 19.9
	Females	31.5 (30.0–37.1)	30.4 (27.1–33.6)	0.18	3.1	−1,7, 7.9
12-item SF survey—MCS	Males	57.3 (55.0–60.3)	52.4 (40.1–57.0)	0.20	5.4	−2.6, 13.5
	Females	49.5 (46.7–54.6)	37.5 (31.0–49.3)	**0.02**	9.2	0.6, 17.8
Pain intensity (a.u.)	Males	2.8 (2.1–3.8)	5.5 (3.6–6.4)	**0.02**	−2.3	−3.9, −0.8
	Females	3.3 (2.7–5.3)	3.8 (3.0–5.0)	0.84	0.01	−1.1, 1.2
Number of pain areas	Males	2.5 (1.2–3.0)	3.0 (2.0–3.0)	0.54	−0.3	−1.6, 0.9
	Females	3.0 (2.0–4.0)	3.0 (3.0–4.0)	0.52	−0.2	−1.1, 0.7
Pain extension (pixels)	Males	18,064 (14,695–30,106)	29,190 (14,158–52,055)	0.46	−7,132	−34,641, 20,375
	Females	29,466 (14,730–43,103)	26,663 (6,499–44,091)	0.52	2,724	−17,373, 22,823
Body mass index (kg/m^2^)	Males	29.7 (26.3–30.2)	28.8 (27.2–32.7)	0.58	−1.6	−5.3, 2.2
	Females	32.0 (26.2–34.7)	31.6 (29.4–39.5)	0.20	−3.4	−7.7, 1.0
Body fat %	Males	27.6 (25.3–30.5)	28.7 (25.5–36.2)	0.54	−2.7	−7.9, 2.5
	Females	40.3 (32.0–42.8)	39.4 (37.7–44.0)	0.29	−2.6	−7.9, 2.8
Fat mass index (kg/m^2^)	Males	8.3 (7.6–8.7)	7.7 (7.1–12.0)	0.86	−1.3	−3.9, 1.4
	Females	12.7 (8.3–14.3)	12.2 (11.0–16.5)	0.64	−2.5	−6.5, 1.5
Waist circumference (cm)	Males	95.8 (93.4–100.3)	97.9 (95.0–109.8)	0.54	−4.8	−14.9, 5.3
	Females	110.2 (98.0–113.7)	111.2 (103.9–118.4)	0.22	−6.7	−16.9, 3.4
Appendicular lean mass index (kg/m^2^)	Males	8.4 (8.2–8.5)	8.1 (8.0–8.5)	0.36	0.05	−0.7, 0.8
	Females	6.6 (6.0–7.3)	6.8 (6.4–7.9)	0.36	−0.6	−1.5, 0.3
Fast walking speed (m/s)	Males	1.4 (1.3–1.5)	1.0 (0.6–1.1)	**0.001**	0.5	0.2, 0.7
	Females	1.3 (1.2–1.5)	0.9 (0.7–1.1)	**0.0001**	0.5	0.3, 0.6
Stair climb test (time—s)	Males	15.1 (13.2–15.6)	30.0 (20.4–44.2)	**0.0001**	−17.6	−27.6, −7.6
	Females	18.4 (16.6–22.9)	39.0 (35.1–44.0)	**0.0001**	−19.8	−29.2, −10.5
30-s chair stand test (repetitions)	Males	11.0 (10.0–11.0)	6.5 (6.0–9.5)	**0.001**	3.2	1.6, 4.9
	Females	10.0 (9.0–11.0)	9.0 (6.0–12.0)	0.28	1.3	−0.7, 3.3

Statistically significant differences are highlighted in bold. CI, confidence interval; 12-item SF survey-PCS, 12-item short form survey-physical component score; 12-item SF survey-MCS, 12-item short form survey-mental component score; CIRS, cumulative illness rating scale.

Significant subgroup differences were observed in male and female patients for different SF-12 components: in fact, the physical quality of life for males and the mental quality of life for females were significantly lower in the high severity subgroup of patients with respect to the low severity subgroup.

In male patients the high severity subgroup showed higher pain intensity, comparable pain extension, and comparable number of pain areas compared to the low severity subgroup, while in female patients the high severity subgroup showed no differences for the three pain phenotype variables with respect to the low severity subgroup.

No subgroup differences were observed for the investigated variables of body size and composition in either male or female patients, while significant differences were observed in physical performance for all variables in males and for two of the three variables (i.e., fast walking speed and 30-s chair stand test performance) in females. Briefly, the physical performance was significantly lower in the high severity subgroup of both males and females with respect to the low severity subgroup.

## 4 Discussion

This is the first study phenotyping patients with late-stage osteoarthritis scheduled for total knee arthroplasty with traditional physical performance tests in combination with innovative digital anthropometric and pain drawing assessments. The main results of this study can be summarized as follows: (i) body size and composition assessment through a digital anthropometric approach was feasible in most of the patients (those presenting no or minimal valgus deformity and who were able to maintain the standing position during the acquisition of the frontal and lateral images): an increased (central) adiposity was observed in almost all patients of both genders, while the appendicular lean mass was low in some male patients; (ii) pain intensity and affective interference were between mild and moderate, while pain activity interference was between moderate and severe in most of the patients; (iii) pain had an anterior distribution (in the periarticular region) in all patients, while several patients of both genders showed also a posterior distribution; (iv) half of the male patients and more than two-thirds of the female patients showed a bilateral knee pain; (v) a relevant impairment of physical performance was observed in all patients: physical performance variables were significantly lower in the high severity subgroups of males and females with respect to the low severity subgroups.

The pre-operative assessment of comorbidities (e.g., obesity, frailty) and other factors (e.g., type of surgery, physical performance) is widely used in the clinical practice to predict the peri-operative risks. For example, in case of body mass index (BMI) in the range 30–40 kg/m^2^, a patient is classified as having a mild systemic disease (according to the ASA Physical Status Classification System) (42) that may contribute to increase the peri-operative risk. Although BMI is a globally applied phenotypic descriptor of adiposity at the population level, its limitations for assessing weight and predicting excess body fat at the individual level are well known (43–46). Consistently, previous (38) and our body composition charts showed that a given BMI embraces a wide range of percentage fat. Thus, previous and present data suggest that moving beyond BMI is required to precisely characterize the health status of individual patients. This is the first study, to our knowledge, showing in a real-world setting the feasibility of a digital anthropometric approach to quantify and characterize the increased adiposity in patients with late-stage knee osteoarthritis. The observation that excess adiposity was associated with an increased abdominal circumference (i.e., a marker of visceral adiposity) in both male and female patients is in agreement with recent studies showing that body shape changes (i.e., increased “apple-shaped” phenotype due to a body weight “shift” from the lower to the upper body, especially to the waist) occurred over the last decades in both men and women of American (47) and European (10) populations. Thus far, smartphone camera technology has only been used to estimate percent body fat and only in academic research settings. To our knowledge, this study is the first investigating in a real-world setting (and through an innovative digital approach) the appendicular lean mass (i.e., a proxy of muscle mass) that was normal in female patients and reduced in some male patients. On the basis of our findings, we suggest that the pre-operative prognostic assessments of patients with late-stage knee osteoarthritis could be improved in the clinical practice through the acquisition of “e-tape” measurements of body size and composition (such as waist circumference, fat mass index, and appendicular lean mass index) that can be more informative and acceptable compared to standard anthropometric measurements (based on weight and height assessment or on the identification of anatomical landmarks through observation and palpation). However, the Mobile Fit app adopted in the present study failed to capture the frontal image in some patients presenting grade II or III valgus deformity: therefore, the avatars of these patients were not available. Methodological implications of this observation are twofold. First, the algorithms adopted to extract the frontal silhouette (that is required in combination with the lateral silhouette to generate the three-dimensional avatar) need to be improved for the use of this app in patients with anatomical limb deformities. Second, three-dimensional scanners could be required to evaluate patients presenting with varus or valgus deformities. It may be hypothesized that the technology of three-dimensional scanners is less sensitive to limb deformities compared to mobile digital anthropometry applications (using photographic images of static subjects, either from anterior and lateral or anterior and posterior views).

The demonstration that patients with late-stage knee osteoarthritis scheduled for total knee arthroplasty showed moderate to severe pain and interference confirms the need to optimize the pre-operative management of pain in this patient population. Although the “*pro re nata*” (as required) prescription of analgesics is commonly used for non-malignant musculoskeletal pain, it may be recommended to adopt a fixed schedule of pharmacotherapy in selected patients. For example, in patients presenting with long duration and high extension (e.g., antero-posterior and/or bilateral distribution) of pain, a fixed-interval analgesic regimen may be more effective than an on-demand schedule in controlling pain intensity, reducing pain interference, and preventing both the spread of pain beyond the initial site as well as symptoms of central sensitization, such as allodynia or hyperalgesia.

The demonstration that patients with late-stage knee osteoarthritis scheduled for total knee arthroplasty showed a relevant impairment of physical performance and that this impairment was associated with perceived disability confirms previous findings (48, 49). Possible mechanisms underlying this impairment include the age-related and sedentary behavior-related (i.e., disuse-related) changes in muscle composition (50–52) and mass (that we found reduced in some male patients), increased adiposity-related intramuscular fatty infiltration (53) and altered skeletal muscle energetics (54), and pain-induced adaptations of the neuromuscular system (55–57).

### 4.1 Limitations

This study has a few limitations that warrant consideration. First, the use of a convenient sample may produce results that cannot be generalized to the target population because of possible selection bias due to the under-representation of subgroups in the sample in comparison to the population of interest. Second, we studied, through a single-centre study design, patients with late-stage osteoarthritis, thus our results cannot be generalized to other centers and to all patients with knee osteoarthritis. Third, the number of patients included was relatively small, thus negative sta­tistical analyses could be a result of type II error and the statistical power of the cluster analysis could be limited. Fourth, body composition estimates obtained by digital anthropometry were not compared with estimates obtained through gold-standard approaches (i.e., four-compartment model for the body fat percentage estimation and whole-body magnetic resonance imaging for appendicular lean mass estimation). Fifth, we did not control for factors that might influence body composition (such as meal timing and hydration status) and might thus add variability to the body composition estimates. Sixth, we cannot infer causality given the cross-sectional design of the study and given the exploratory nature of the cluster analysis: it remains unclear whether the increased adiposity (and/or low appendicular lean mass observed in some male patients) and/or localized pain can contribute to the observed impairment in physical performance and whether the physical performance impairment can contribute to (or can result from) perceived disability. Consequently, future longitudinal investigations are required to determine whether the factors identified in this study (i.e., increased central adiposity, low appendicular lean mass, and impaired physical performance) can be used as prognostic indicators for poor postoperative outcome following knee arthroplasty.

## 5 Conclusion

The present study investigated body size and composition variables, pain characteristics, and physical performance variables in patients with late-stage osteoarthritis scheduled for total knee arthroplasty. We found that body size and composition assessment through a digital anthropometric approach was feasible in most of the patients: an increased (central) adiposity was observed in almost all patients, while some male patients showed also a reduced appendicular lean mass. Pain showed an anterior distribution in all patients and half of the male patients and more than two-thirds of the female patients showed a bilateral knee pain. We also found in all patients a moderate to severe pain and interference and a relevant impairment of physical performance. We suggest that innovative digital tools can be used to quantify the changes in body size and composition and the pain location and extension in patients with late-stage knee osteoarthritis.

## Data Availability

The raw data supporting the conclusions of this article will be made available by the authors, without undue reservation.

## Supplementary material

Supplementary Figure S1 Bar charts of the final cluster centers for male patients (left panel) and female patients (right panel). Blue columns: Lequesne index; green columns: lower extremity function T-score; light brown columns: Knee Society functional abilities score; purple columns: activity pain interference score; yellow columns: affective pain interference score. The higher the Lequesne index and the pain interference scores, the higher the perceived disability, while the lower the Knee Society functional abilities score and the lower extremity function T-score, the higher the perceived disability.
