# Supplementary material for: Innovative digital approaches to characterize core factors of patients with late-stage knee osteoarthritis: a cross-sectional study
Source: Front Digit Health. 2026 Jan 13;7:1709182. doi: 10.3389/fdgth.2025.1709182 (PMC12835352; doi:10.3389/fdgth.2025.1709182)
Supplement: Supplementary Figure S1 — Bar charts of the final cluster centers for male patients (left panel) and female patients (right panel). Blue columns: Lequesne index; green columns: lower extremity function T-score; light brown columns: Knee Society functional abilities score; purple columns: activity pain interference score; yellow columns: affective pain interference score. The higher the Lequesne index and the pain interference scores, the higher the perceived disability, while the lower the Knee Society functional abilities score and the lower extremity function T-score, the higher the perceived disability. [file Datasheet1.pdf]

Supplementary Material

VARIABLE	*EQUATION*	*REF*
Body fat (%)<br>***- abdomen circ. <103.5 cm***	*48.837 – 7.2745 × (1 for male, 0 for female) + 0.46929 × (right thigh circ.) – 17.387 x* $(\frac{right\ bicep\ circ.\ +\ left\ bicep\ circ.\ +\ right\ calf\ circ.\ +\ left\ calf\ circ.\ +\ right\ thigh\ circ.\ +\ left\ thigh\ circ.}{maximum\ stomach\ circ.})$	***1***
Body fat (%)<br>***- abdomen circ. >103.5 cm***	*-1.1789 – 3.5143 × (1 for male, 0 for female) + 0.53795 × (abdomen circ.) – 0.001076 × (body surface area)*	***1***
Appendicular lean mass (kg)<br>***- male subject***	*-27.386 + 2.566 × (1 for NHOPI race, 0 for other races) + 0.132 × height + 0.221 × weight + 0.068 × (head circ.) + 0.087 × (chest circ.) + 0.212 × (forearm circ.) - 0.038 × (waist circ.) + 0.020 × (thigh circ.) - 0.024 × (outside leg length) + 0.0008 × (surface area arm) + 0.00005 × (arm volume) - 0.0002 × (torso volume) + 0.0003 × (leg volume)*	***2***
Appendicular lean mass (kg)<br>***- female subject***	*-12.622 – 0.023 × age – 0.191 × (1 for Caucasian race, 0 for other races) + 0.427 × (1 for Black race, 0 for other races) - 0.288 × (1 for Hispanic race, 0 for other races) + 0.553 × (1 for NHOPI race, 0 for other races) - 0.709 × (1 for Other race, 0 for Caucasian, Black, Asian, Hispanic, NHOPI, American Indian races) + 0.088 × height + 0.135 × weight + 0.001 × (head circ.) + 0.019 × (collar circ.) + 0.048 × (forearm circ.) + 0.078 × (upper arm circ.) - 0.039 × (waist circ.) + 0.083 × (ankle circ.) + 0.023 × (outside leg length) + 0.0003 × (surface area leg)*	***2***

Supplementary table 1.
Prediction equations for the body fat percentage and appendicular lean mass.
Circ.= circumference; NHOPI: Native Hawaiian and other Pacific Islander.
Units of measurement: cm for all circumferences and lengths; cm^2 for surface areas; cm^3 for volumes; kg for weight; years for age.

1. Graybeal AJ, Brandner CF, Tinsley GM. Visual body composition assessment methods: a 4-compartment model comparison of smartphone-based artificial intelligence for body composition estimation in healthy adults. *Clin. Nutr.* (2022) 41:2464-72. doi: 10.1016/j.clnu.2022.09.014
2. McCarthy C, Tinsley GM, Yang S, Irving BA, Wong MC, Bennett JP, et al. Smartphone prediction of skeletal muscle mass: model development and validation in adults. *Am J Clin Nutr.* (2023) 117:794-801. doi: 10.1016/j.ajcnut.2023.02.003
