# Supplementary figures and images for: Innovative digital approaches to characterize core factors of patients with late-stage knee osteoarthritis: a cross-sectional study
Source: Front Digit Health. 2026 Jan 13;7:1709182. doi: 10.3389/fdgth.2025.1709182 (PMC12835352; doi:10.3389/fdgth.2025.1709182)

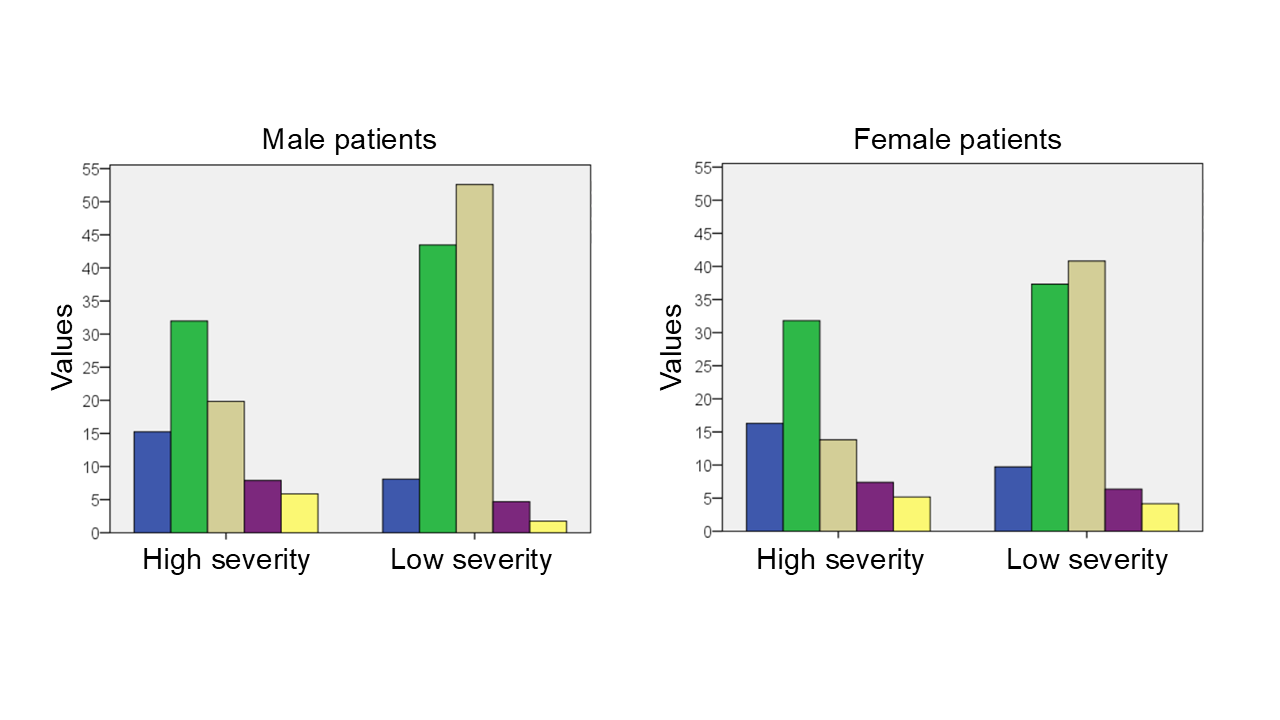

Supplement: Supplementary file 2 [file Image1.tif]
